# *TBK1* and *TNFRSF13B* mutations and an autoinflammatory disease in a child with lethal COVID-19

**DOI:** 10.1038/s41525-021-00220-w

**Published:** 2021-07-01

**Authors:** Axel Schmidt, Sophia Peters, Alexej Knaus, Hemmen Sabir, Frauke Hamsen, Carlo Maj, Julia Fazaal, Sugirthan Sivalingam, Oleksandr Savchenko, Aakash Mantri, Dirk Holzinger, Ulrich Neudorf, Andreas Müller, Kerstin U. Ludwig, Peter M. Krawitz, Hartmut Engels, Markus M. Nöthen, Soyhan Bagci

**Affiliations:** 1 Institute of Human Genetics, University of Bonn, School of Medicine & University Hospital Bonn, Bonn, Germany; 2 Institute of Genomic Statistics and Bioinformatics, University of Bonn, Bonn, Germany; 3 Neonatology and Pediatric Intensive Care, University of Bonn, School of Medicine & University Hospital Bonn, Bonn, Germany; 4 Department of Pediatric Hematology-Oncology, University of Duisburg-Essen, Essen, Germany; 5 Institute for Medical Biometry, Informatics and Epidemiology, Medical Faculty, University of Bonn, Bonn, Germany; 6 Core Unit for Bioinformatics Data Analysis, Medical Faculty, University of Bonn, Bonn, Germany; 7 Department of Diagnostic and Interventional Radiology, University of Bonn, School of Medicine & University Hospital Bonn, Bonn, Germany

**Keywords:** Infection, Genetic testing, Viral infection, Autoinflammatory syndrome

## Abstract

Among children, severe acute respiratory syndrome coronavirus 2 (SARS-CoV-2) infections are typically mild. Here, we describe the case of a 3.5-year-old girl with an unusually severe presentation of coronavirus disease (COVID-19). The child had an autoinflammatory disorder of unknown etiology, which had been treated using prednisolone and methotrexate, and her parents were half cousins of Turkish descent. After 5 days of nonspecific viral infection symptoms, tonic-clonic seizures occurred followed by acute cardiac insufficiency, multi-organ insufficiency, and ultimate death. Trio exome sequencing identified a homozygous splice-variant in the gene *TBK1*, and a homozygous missense variant in the gene *TNFRSF13B*. Heterozygous deleterious variants in the *TBK1* gene have been associated with severe COVID-19, and the variant in the *TNFRSF13B* gene has been associated with common variable immunodeficiency (CVID). We suggest that the identified variants, the autoinflammatory disorder and its treatment, or a combination of these factors probably predisposed to lethal COVID-19 in the present case.

## Introduction

Coronavirus disease (COVID-19) is an infectious disorder caused by severe acute respiratory syndrome coronavirus 2 (SARS-CoV-2). Among children, severe COVID-19 disease courses are rare, and mainly affect children with underlying medical conditions^1–3^. Underlying medical conditions reported to date in children with severe COVID-19 include chronic lung disease, obesity, neurological and developmental disorders, and immunosupressed status^3^.

Recent research in adult SARS-CoV-2 cases has identified associations between severe COVID-19 and several rare genetic variants in genes implicated in TLR3-, TLR7-, and IRF7-dependent IFN type I immunity, including heterozygous variants in the gene *TBK1*^4–7^.

The present report describes the case of a 3.5-year-old girl with an unclassified autoinflammatory disease who died secondary to COVID-19. This severe COVID-19 disease course is unusual in a child of this age. We therefore suspected an underlying inborn disorder, and conducted trio exome sequencing. This identified one variant respectively in the genes *TBK1* and *TNFRSF13B*. These represent potential predisposing genetic factors for severe COVID-19, in the context of the unclassified autoinflammatory disease and its treatment regimen.

## Results

### Case report (for further details, see Supplementary Note)

The 3.5-year-old girl was born to consanguineous parents (half cousins with one common grandfather) of Turkish descent. From the age of 6 months, the girl had had a history of an unclassified autoinflammatory disease, whose key features comprised chronic synovitis/polyarthritis, and recurrent episodes of exanthema and systemic inflammation. Immunodiagnostics (vaccination responses and immune status) had been unremarkable, and no recurrent infections had been observed. In the 10 months prior to death, the autoinflammatory disease had been treated with prednisolone (0.16 mg/kg per day) and methotrexate (12.5 mg/m²/week, last administration 3 days prior to hospital admission). The parents reported that for the 5 days prior to admission, the child had displayed nonspecific signs of infection (intermittent fever and ear pain). On day 5, the fever had subsided. However, the girl had become anxious, and visibly distressed by loud noises. Two hours later, the girl had displayed features of a tonic-clonic seizure, which stopped spontaneously after 5 min. The girl was then admitted to hospital, and diagnosed as being in an afebrile postictal state with signs of an acute upper airway infection. In the period 5 to 11 h post-admission, several tonic-clonic seizures occurred. Native emergency computed tomography (CT) and a lumbar puncture were then performed. The CT results were unremarkable. However, lumbar puncture revealed pleocytosis, increased levels of protein and lactate, and a normal glucose level. At 90 min post-lumbar puncture, acute heart failure occurred. After 15 min of cardiopulmonary resuscitation, sufficient cardiopulmonary function resumed. However, the girl remained unconscious. Instrumental diagnostics revealed evidence of cardiac insufficiency, diminished electrical activity of the brain, and bilateral pneumonic infiltrations. Laboratory diagnostics revealed disseminated intravascular coagulation, metabolic acidosis, and elevated levels of creatinine and transaminase. At this time-point, throat swabs were taken for SARS-CoV-2 testing. Two days after hospital admission, signs of multiorgan failure developed. A repeat cranial CT revealed signs compatible with a thrombosis of the dural sinus and venous infarction. A positive SARS-CoV-2 test result was then returned. Despite continued intensive care management, the girl died of multi-organ insufficiency 62 h post-hospital admission.

### Exome sequencing

For a child, the girl experienced an unusually severe COVID-19 course. We therefore suspected an underlying inborn disorder, and performed trio exome sequencing. In total, we identified around 112,000 variants. Of these, 800 had allele frequencies below 2% and showed some evidence for deleteriousness (Table 1). Further prioritization strategies focused on variants that: (1) were located in genes of potential relevance to the phenotype; and (2) segregated in compliance with a fully penetrant Mendelian disease model. Seven variants fulfilled both criteria (Supplementary Data 1). Of these, one homozygous variant in the genes *TBK1* and *TNFRSF13B*, respectively (see Table 2) were considered likely causal factors for the severe COVID-19 disease course observed in the present case.

**Table 1:** Variant filtering and analysis scheme.

Step	Number of variants
Trio exome sequencing	111,596
Annotation and filtering with Ensembl VEP	
Filter 1: AF <2 % in population subcohorts, <5 x homozygous occurrences in gnomAD exomes v2.1.1	7,031
Filter 2: VEP impact HIGH or MODERATE or SpliceAI score >0.1	841
Prioritization	
1) Variants in candidate genes of phenotypic relevance	92
2) Variants segregating in compliance with a Mendelian Disease (full penetrance)	65
Variants shared between (1) and (2) (these variants can be found in Supplementary Data 1)	7 (genes: *TTN* (2x), *DNM1L*, *TBK1, TNFRSF13B,* and *RUNX1* (2x))
Variants considered relevant to the severe COVID-19 disease course	2 (genes: *TBK1* and *TNFRSF13B*)

Note that gene symbols are italicized.

*COVID-19* coronavirus disease, *VEP* variant effect predictor, *AF* allele frequency, *IGV* integrative genomics viewer.

**Table 2:** Potentially causative variants identified by exome sequencing.

Gene transcript	HGVS rs-ID	Zyg.	Associated disease (Inheritance)	gnomAD allele count	gnomAD constraint metrics
*TBK1* NM_013254.4	c.1760 + 4_1760 + 7del; p.? rs753802322	hom	Severe COVID-19 (AD); Susceptibility to acute infection-induced encephalopathy 8 (AD); Frontotemporal dementia and/or amyotrophic lateral sclerosis 4 (AD)	1 (het) / 0 (hom)	pLI: 0.08 Missense-Z: 1.9 o/e (LoF): 0.25 (0.16-0.42) o/e (Missense): 0.72 (0.65-0.79)
*TNFRSF13B* NM_012452.2	c.310 T > C; p.(Cys104Arg) rs34557412	hom	Common variable immune deficiency 2 (AD / AR); Immunoglobulin A deficiency 2 (?)	983 (het) / 4 (hom)	pLI: 0 Missense-Z: −1.2 o/e (LoF): 1.73 (1.14-1.96) o/e (Missense): 1.26 (1.13-1.41)

Note that gene symbols are italicized.

*HGVS* sequence variant description in accordance with the recommendations of the human genome variation society, *rs-ID* dbSNP reference single-nucleotide polymorphism number, *Zyg* Zygosity, *hom* homozygous, *het* heterozygous, *AD* autosomal-dominant, *AR* autosomal-recessive, *pLI* probability of loss of function intolerance, *Missense-Z* *Z*-score indicating the depletion of missense variants from a gene, *o/e* observed/expected metric with its 90% confidence interval shown in brackets for loss-of-function (LoF) or missense variants.

### Homozygous variant in the *TBK1* gene

In the *TBK1* gene, a homozygous deletion was identified near the exon-intron border between exon 16 and intron 16 (NM_013254.4:c.1760 + 4_1760 + 7del). Both parents carried the variant in a heterozygous state. Because of the low coverage in the exome data at this locus, this variant was confirmed in the patient DNA using Sanger sequencing (see Supplementary Fig. 1). The variant altered the splice site by deleting four nucleotides (TAAgtaagtatcc, capitalized bases in the exon, crossed-out bases deleted in the index patient). The effect of the variant on the transcriptional level is difficult to ascertain in silico. The deep learning-based tool spliceAI generated a high confidence prediction of a loss of the splice donor site (delta-score/estimated probability (*p*): 0.93; coordinates used for prediction: chr12:64890189AAGTA > A/hg19). However, the software did not predict the possible location of a new splice donor site for this variant, and generated a low confidence level prediction (*p*: 0.06) that the original donor splice site is used. SpliceAI also predicted that complete skipping of the exon might be possible (*p*: 0.34 for a loss of the splice acceptor site of exon 16). This variant is reported in a heterozygous state in one individual in the population-based database gnomAD. However, it is not reported in any other population-based database, or in any phenotype-related database. MutationTaster (mutationtaster.org) classifies the variant as a polymorphism. In contrast, the combined annotation dependent depletion (CADD) score of this variant is 34, indicating that it ranks among the 0.1% most deleterious variants (version 1.4/https://cadd.gs.washington.edu). Sequencing of *TBK1* cDNA from the heterozygous parents revealed the presence of an alternatively spliced *TBK1* transcript, in which exon 16 was skipped (r.1721_1760del, see Supplementary Fig. 1). This is consistent with results from the in silico prediction by SpliceAI. At the protein level, skipping of exon 16 is predicted to result in a frameshift and a premature stop codon, which could result in a truncated TBK1 protein that is characterized by the absence of the entire coiled coiled domain 2 (CCD2) and the partial absence of CCD1 (p.(Arg574Serfs*11)).

### Homozygous variant in the *TNFRSF13B* gene

In the gene *TNFRSF13B*, a homozygous missense variant was identified in exon 3 (NM_012452.2:c.310 T > C;p.(Cys104Arg)). Both parents carried the variant in a heterozygous state. The allele frequency of this variant in population-based databases ranges up to 0.5% (gnomAD European population), and the variant is listed four times in gnomAD in a homozygous state (Table 2). Several mutation prediction programs (MutationTaster, SIFT/PROVEAN/provean.jcvi.org, and PolyPhen-2/genetics.bwh.harvard.edu/pph2/) classify the variant as pathogenic. The CADD score of the Cys104Arg variant is 26.1, indicating that it ranks among the 1% most deleterious variants.

## Discussion

The unusually severe and ultimately lethal COVID-19 disease course observed in the present preschool child suggested the presence of predisposing factors, such as an inborn error of immunity. In summary, we identified the following potential predisposing factors:

The homozygous variant in *TBK1* (NM_013254.4:c.1760 + 4_1760 + 7del) had a demonstrable effect on splicing (r.1721_1760del). Potentially, this variant could result in a truncated TBK1 protein (p.(Arg574Serfs*11)) that lacks parts of CCD1 and the entirety of CCD2. Research has shown that the deletion of CCD1 results in a dysfunctional TBK1 protein^8^. The *TBK1* gene is involved in the TLR3 and IRF7 dependent IFN type I pathway, in which an excess of deleterious variants—including two heterozygous *TBK1* mutations— has been associated with a severe COVID-19 disease course^4^. However, available data on the role of the type I IFN pathway in COVID-19 severity are conflicting. While two recent preprints concerning exome-sequencing data failed to replicate the association between rare variants in this pathway and severe COVID-19^9,10^, three genetic studies have found an association between variants in the gene *TLR7*—which also activates type I IFN transcription—and a severe COVID-19 disease course^5–7^.

Type I IFNs display strong anti-SARS-CoV-2 activity in cultured cells^11^, and the virus itself inhibits numerous proteins of the innate IFN type I response^12^. Research in a murine knockout model demonstrated that TBK1 is important in terms of the interferon response of embryonic fibroblasts to events such as viral infections^13^.

A recent report suggested that a homozygous truncating variant of *TBK1*, c.1318 C > T;p.(Arg440*), might have been the causal factor for an autoinflammatory disease in a preschool child. The girl had presented with a history of recurrent fever and erythematous rash, growth failure, and neurocognitive retardation^14^.

Overall, a plausible hypothesis is that the present *TBK1* variant caused an untuned IFN type I response, which led in turn to a propensity to a severe COVID-19 disease course. Additionally, homozygous *TBK1* variants may cause an autoinflammatory disorder, as observed in both the present case and the previously reported preschool child. Further studies are warranted to elucidate the potential contribution of the *TBK1* variant to both the severe COVID-19 disease course, and the underlying autoinflammatory disorder, observed in the present case.

In *TNFRSF13B* we identified a homozygous missense variant (NM_012452.2:c.310 T > C;p.(Cys104Arg)). Variants in the gene *TNFRSF13B* have been associated with the common variable immunodeficiency (CVID) syndrome “Common variable immune deficiency 2” (CVID2, OMIM # 240500), and the immunoglobulin A deficiency (IGAD) “Immunoglobulin A deficiency 2”/IGAD2 (OMIM # 609529). The present variant is listed several times in phenotype-related databases (ClinVar/ncbi.nlm.nih.gov/clinvar, HGMD/hgmd.cf.ac.uk, and LOVD/lovd.nl). Interpretations of its clinical significance diverge, and vary from probably benign to pathogenic with “likely pathogenic” or “pathogenic” as the most common interpretations. Functional studies have demonstrated that the variant has a damaging effect on protein function^15–17^, and research has demonstrated B-cell dysfunction in mice that are transgenic for the murine homolog of the variant^18,19^.

CVID is characterized by hypogammaglobulinemia, with decreased levels of IgG and a marked decrease in at least one of the isotypes IgM and IgA (https://esid.org/Education/Common-Variable-Immunodeficiency-CVI-diagnosis-criteria). Splenomegaly, lymphoid hyperplasia, and autoimmune rheumatological diseases, such as juvenile forms of chronic inflammatory arthritis, may also occur^20^. Research suggests that CVID2 could have both a recessive and a dominant mode of inheritance^15^. Additionally, reduced penetrance and variable expressivity have been observed in homozygous carriers of the reported *TNFRSF13B* variant for CVID2. Some homozygous carriers are asymptomatic, or show immunological abnormalities that do not meet the clinical criteria for CVID^17,21,22^. The present patient did not fulfill the clinical criteria for CVID at disease onset, since she did not display hypogammaglobulinemia, had an intact vaccination response, and had no apparent history of recurrent infections. However, this may have been attributable to a reduced penetrance of the CVID-phenotype, which may have been age-related. A literature search revealed a total of 18 CVID patients with COVID-19, two of whom died^23–27^. The largest study of comorbid CVID/COVID-19 to date included ten patients. Due to the relatively mild disease course observed in all cases, the authors concluded that this population showed no increased risk for a severe COVID-19 disease course^26^. In summary, the *TNFRSF13B*-variant may have contributed to the fulminant SARS-CoV-2 infection and to the nonspecific, preexisting autoinflammatory disease observed in the present case.

COVID-19 disease progression may have been exacerbated by the child’s preexisting unclassified autoinflammatory disease and treatment with prednisolone and methotrexate. A recent study analyzed 3729 patients with inflammatory rheumatic diseases^28^, and found that the use of high dose glucocorticoids (≥10 mg/day; odds ratio (OR) 1.69) and high disease activity (OR 1.87) were associated with a higher probability of death secondary to COVID-19. In contrast, methotrexate monotherapy was associated with a lower probability of death compared to the absence of a disease-modifying anti-rheumatic drug (DMARD) treatment (OR 0.47). Therefore, the child’s prednisolone treatment (0.16 mg/kg per day) in particular may have promoted the severe COVID-19 disease course. However, the referenced study involved adult patients, and the issue of whether the findings can be generalized to pediatric populations remains unclear.

The rapid deterioration observed in the present case may have been attributable to complications of the SARS-CoV-2 infection, such as a dural sinus thrombosis which may have been overlooked by the primary non-contrast CT investigation^29^. Another potential complication may have been a SARS-CoV-2 infection of the central nervous system that escaped detection during CSF testing. The recently reported multisystem inflammatory syndrome in children (MIS-C) is another SARS-CoV-2 associated complication for which fatalities have been reported^30^. While we cannot exclude MIS-C the child did not display some of the typical symptoms of MIS-C, such as persistent pyrexia, gastrointestinal symptoms, and mucocutaneous involvement^31^. Furthermore, the abrupt onset of severe COVID-19 symptoms during active infection is more suggestive of an immune deficiency than the hyperinflammation that is frequently seen in MIS-C^31^. In addition, the child had blood group A, which is a reported risk factor for SARS-CoV-2 susceptibility^32^.

We describe three potential predisposing factors—a homozygous splice site mutation in *TBK1*, a deleterious missense mutation in *TNFRSF13B*, and the presence of an autoinflammatory disease and its treatment regimen—which might have promoted the severe disease course observed in the present case either alone or in concert. At writing, we are unable to assign the contribution of each factor with certainty. In our opinion, however, the currently available evidence favors the *TBK1* variant over the other two factors as a driver of the severe COVID-19 disease course. In view of the recent report of a child with an autoinflammatory disease and a homozygous nonsense variant in *TBK1*^14^, the present *TBK1* variant might also have been the causal factor for the child’s preexisting, unclassified autoinflammatory disease.

In general, our data suggest that genetic testing of children with severe COVID-19 is warranted in order to identify monogenic factors, such as defects in type I IFN defense, which may drive a severe disease course. This could identify groups of individuals who are particularly susceptible to severe COVID-19, and who might benefit form special protective measures or specific therapeutic approaches.

## Methods

### Human participants

Prior to the molecular analyses, written informed consent was obtained from both parents, including consent to the potential scientific use of the results. After the analyses were completed, the results were discussed with the parents and the parents agreed to a publication as a case report. Our case report is a retrospective report of a clinical case and its diagnostic work-up. Thus, the report is not part of a planned scientific study. The relevant ethics committee of the University of Bonn was informed about the planned publication of the case report and has approved the procedure.

### Trio exome sequencing

Exome sequencing was conducted at the Institute of Human Genetics of the University of Bonn as part of the routine diagnostic work-up. DNA was extracted from peripheral blood leukocytes. All coding genomic target regions (exome), as well as the adjacent intronic regions, were enriched using the SureSelect Human All Exon V6 Enrichment Kit (Agilent Technologies, Santa Clara, California). These were sequenced on a NextSeq system (Illumina, San Diego, California) as 2 × 100 bp paired-end reads. Alignment to the reference genome GRCh37/hg19 was performed using the Burrow Wheeler Aligner software (BWA version 0.7.17-r1188). Subsequent variant calling was performed in accordance with the “GATK Best Practice” guidelines (GATK version 4.1.3.0). In total, 90% of the target regions were covered at least 20-fold, and 94% were covered at least tenfold.

The filtering and prioritization strategy is outlined in Table 1. Annotation and filtering were performed using Ensembl Variant Effect Predictor (VEP, version 97, www.ensembl.org/info/docs/tools/vep/). First, filtering was performed for variants with an allele frequency of <2% in subcohorts of the population-based databases Genome Aggregation Database (gnomAD, v2.1.1); Exome Variant Server (EVS); and 1000Genomes; and with <5 reported homozygous occurrences in gnomAD. Next, filtering was performed for variants with a high or moderate impact—as predicted by VEP—or with a spliceAI-score of >0.1 in any category (https://github.com/Illumina/SpliceAI). This corresponds to non-synonymous single nucleotide variants; insertions and deletions (indels); and variants with potential effects on splice sites. To prioritize variants in genes of relevance to the present phenotype, a gene list was compiled, which was mainly based on terms from the Human Phenotype Ontology (HPO), and gene panels from the Genomics England PanelApp (https://panelapp.genomicsengland.co.uk/). The list of genes is provided in Supplementary Data 1. To prioritize variants with a recessive or dominant inheritance pattern, a filter for high quality variants (QD >2.0 and FS <60.0 and MQ >40.0 and MQRankSum > −12.5 and ReadPosRankSum > −8.0) was first applied. Homozygous, compound heterozygous, and potential de novo variants were then identified using the tool VASE (version 0.24, https://github.com/david-a-parry/vase). Potential de novo variants underwent additional filtering to ensure a genotype quality (GQ) of >50 in each individual. Variants were assessed by at least two independent exome evaluators, who evaluated the variants on the basis of estimated pathogenicity following the ACMG/AMP guidelines; segregation; potential relevance to the phenotype; and the appearance of raw sequencing data (see also Supplementary Data 1). As a complementary analysis, human leukocyte antigen (HLA) typing was performed using exome data (see Supplementary Note and Supplementary Data 1). The *TBK1* variant was confirmed at the genomic DNA-level using standard Sanger sequencing (F-primer: TGGGCCATAAGTAACCAATGAG and R-primer: GGGAGCAGATGTGTGAACCT).

### Transcript analysis

Blood samples were collected from the parents using the PaxGene Blood RNA Kit (PreAnalytiX, Switzerland), and RNA was extracted in accordance with the manufacturer’s protocol. RNA was quantified using a Nanodrop 2000 spectrophotometer (Thermo Fisher Scientific, Waltham, Massachusetts), and reverse transcribed into cDNA using SuperScript II and oligodT primers. A cDNA fragment was amplified using the primers AGGCACTCATCCGAAAGACA and CCACCCCTTCCATCTCTTCC, which are located in *TBK1* exons 14 and 20, respectively. Sanger sequencing was performed using BigDye v 3.1 (Thermo Fisher Scientific). Subsequent capillary electrophoresis was performed on an ABI 3130XL device (Thermo Fisher Scientific). Data analysis was performed using SeqMan (DNAStar Inc, Madison, Wisconsin).

### Reporting summary

Further information on research design is available in the Nature Research Reporting Summary linked to this article.

## Supplementary information

Supplementary Information

Supplementary Data 1

Reporting Summary

## Data Availability

A list of the genes that were prioritized for exome analysis is available in Supplementary Data 1, and the variants with the highest prioritization in our approach are provided in Supplementary Data 1. The chromatogram files underlying Supplementary Fig. 1 are available upon request. The full exome data set cannot be shared due to a lack of parental consent. Variants were submitted to ClinVar, and are available under accession codes VCV000005302 and VCV001050801.

## Code availability

No previously unreported computer code or algorithm was used. Data analysis was performed using standard tools, as described.
