# Supplementary material for: TBK1 and TNFRSF13B mutations and an autoinflammatory disease in a child with lethal COVID-19
Source: NPJ Genom Med. 2021 Jul 1;6:55. doi: 10.1038/s41525-021-00220-w (PMC8249618; doi:10.1038/s41525-021-00220-w)
Supplement: Supplementary file 1 — Supplementary Information [file 41525_2021_220_MOESM1_ESM.pdf]

# Supplementary information

Supplement to: Schmidt A, Peters S, Knaus A, et al.

**Table of contents**

**Supplementary Notes** ........ 3

**Detailed clinical report** ........ 3

**Human leukocyte antigen (HLA) typing from exome data** ........ 8

**Description of Supplementary Data** ........ 8

**Supplementary Figure 1** ........ 9

**Supplementary References** ........ 10

## Supplementary Notes

## Detailed clinical report

The 3.5 year old girl had a history of an unclassified autoinflammatory disorder, which had commenced at the age 6 months and which was characterized by chronic synovitis / polyarthritis, and recurrent episodes of exanthema and systemic inflammation. Previous laboratory testing had revealed persistent mild hypochromic microcytic anemia, thrombocytosis, and elevated C-reactive protein (CRP). Immunodiagnostics (vaccination responses and immune status) had been unremarkable, and genetic testing (chromosomal analysis, chromosomal microarray, multigene panel for autoinflammatory diseases and for interferonopathies (genes assessed: *ADA2*, *ELANE*, *IL1RN*, *IL36RN*, *LPIN2*, *NLRC4*, *NLRP12*, *NLRP3*, *NOD2* and *PSTPIP1*; *TREX1*, *SAMHD1*, *STING* and *CECR1*) had failed to establish any definite diagnosis. In the 10 months prior to admission, therapy with methotrexate (12.5 mg/m²/week, last administration 3 days before hospital admission) and prednisolone (0.16 mg/kg per day) had been initiated with a good clinical response and a resolution of the arthritis. No other diseases or developmental abnormalities had been observed, and the girl had no previous history of seizures. The parents were half cousins (one common grandfather) of Turkish descent. Two of the girl's female paternal second cousins had a reported history of autoimmune arthritis, which had been treated with methotrexate and etanercept.

In the 5 days prior to hospital admission, the girl had displayed nonspecific signs of infection. The parents reported that on the first day of illness, she had had a runny nose and a fever of around 38 °C, and had complained of ear pain. On the second day of illness, the girl had attended a routine, scheduled appointment with the girl's resident pediatrician for methotrexate treatment monitoring. The pediatrician had documented the girl's general

condition as good, with signs of a minor, nonspecific viral infection without pyrexia. The regular dose of methotrexate had therefore been administered. The parents reported that in the night of the third to the fourth day of illness, the girl had developed a fever of up to 39 °C. On the subsequent morning (fourth day of illness), the girl had been assessed at a pediatric outpatient clinic. No fever was detected, and a diagnosis of a minor, nonspecific viral infection was assigned. During the fourth and fifth days of illness, no fever was noted by the parents. Over the five days of illness, no cough, respiratory distress, vomiting, or diarrhea had occurred.

The parents reported that on the afternoon of the fifth day of illness, the girl had become anxious and visibly distressed by loud noises. Around 2 hours later, the girl was incontinent of urine, her eyes rolled backwards, and her arms and legs began to jerk violently. These signs stopped without intervention after 5 minutes. The girl was then taken to a local general hospital for assessment.

On admission, the girl was noted to be in a postictal state with no focal neurological deficits. Physical examination revealed normal skin color and turgor, pulse, respiratory rate, and blood pressure. The girl showed signs of an acute upper airway infection (pharyngeal erythema, but no rhinitis). The core body temperature was 36.6 °C, the pupils were photoreactive, and no clinical signs of meningitis were observed. Hematological evaluation revealed an increased CRP (98.2 mg/L); leukopenia (5.2 per nL); anemia (7.3 g/dl); and thrombocytopenia (112 per nL). Biochemical evaluation revealed normal levels of creatinine; alanine transaminase (ALT); aspartate transaminase (AST); creatinine kinase (CK); lactate dehydrogenase (LDH); and gamma-glutamyltransferase (GGT). The girl was hospitalized for further assessment and clinical observation, and supportive oxygen supplementation was administered. In the period 5 to 11 hours post-admission, several non-febrile, generalized tonic-clonic seizures occurred, which responded to treatment with phenobarbital and benzodiazepines. An emergency native

computed tomography (CT) was performed 9 hours post-admission, which revealed no evidence of brain edema or intracranial hemorrhage. Treatment with cefotaxime and acyclovir was commenced, and a lumbar puncture was performed. Analysis of the cerebrospinal fluid (CSF) revealed 16 cells/μl (3 neutrophils), elevated levels of protein (272 mg/dl); and lactate (3.1 mmol/l) and a normal level of glucose (71 mg/dl). Polymerase chain reaction testing of the CSF for Borrelia, Enterovirus, Herpes simplex virus (HSV) 1+2, and mycoplasma was performed. All tests were negative. One hour post lumbar puncture, the girl developed pyrexia (39 °C) and acute respiratory distress requiring 2 liters of nasal oxygen. Ibuprofen was administered to counteract the pyrexia. At 90 minutes post-lumbar puncture (13 hours post-admission), a sudden drop in heart rate (< 30/min) and transcutaneous oxygen saturation (< 70%) occurred. Cardio pulmonary reanimation (CPR) was therefore initiated. This comprised mask ventilation; subsequent intubation and cardiac massage; repeated doses of adrenaline (intravenous and then intratracheal following intubation); circulatory volume restitution; and administration of sodium bicarbonate. After approximately 15 minutes of CPR, the heart rate and oxygen saturation normalized. Capillary blood gas-testing revealed an acidosis (pH of 6.9). The girl was then transferred to the hospital's pediatric intensive care unit (PICU). Echocardiography revealed moderate contractility and a low ejection fraction, and treatment with dobutamine was therefore initiated. Due to a pronounced and persistent metabolic acidosis, repeated administration of sodium bicarbonate was required. Hematological laboratory analyses revealed signs of disseminated intravascular coagulation (Quick 19%, PTT 50.9 sec, Fibrinogen 78 mg/dl, AT-III 27%, D-Dimers >20 μg/ml); anemia (8.1 g/dl); and thrombocytopenia (111x10*9/l). Biochemistry analyses revealed elevated levels of creatinine (1.18 mg/dl); ammonia (157 μg/dl); LDH (2229 U/l); CK (6892 U/l); creatine kinase myocardial band (CK-MB) (645 U/l); AST (1397 U/l); and ALT (476 U/l). Erythrocyte concentrates, fresh frozen plasma, antithrombin III, and vitamin K were

administered. Four hours post-PICU admission (approx. 18 hours post-initial hospital admission), the child's core body temperature was 34 °C. An amplitude integrated electroencephalography (aEEG) investigation revealed a burst suppression and flat trace pattern, which remained unchanged even after normalization of the body temperature using a warming device. Despite the lack of any form of sedation, the girl showed no reaction to pain stimuli. The pupils were fixed and dilated.

Due to the clinical deterioration and repeated drops in transcutaneous oxygen saturation, a chest X-Ray was performed. This revealed bilateral pneumonic infiltrations. On the basis of a suspected diagnosis of aspiration pneumonia, ampicillin was administered. At this time-point, the PICU staff were informed that the father had had contact with a SARS-CoV-2 positive person 4 weeks earlier. A throat swab was therefore taken from the patient for SARS-CoV-2 and influenza testing. Over the subsequent 18 hours, the oxygen requirement increased to 100%, and the clinical status and laboratory parameters continued to deteriorate, indicating a respiratory distress syndrome with multiorgan failure.

The girl was therefore transferred to the PICU at the University Hospital of Bonn for possible extracorporeal membrane oxygenation (ECMO) treatment (approx. 36 hours post-initial admission). On admission, the girl presented with a livid skin color (abdomen, and upper and lower extremities); a recapillarization time of 3 - 5 seconds; edema of the hands, feet, and eyelids; a respiratory rate of 40/min; and a core body temperature of 32.9 °C. The child's heart rate was 110/min, and the mean arterial pressure (MAP) was 40 mmHg. Adequate saturation was achieved using Biphasic Positive Airway Pressure (BIPAP) ventilation (settings: peak inspiratory pressure (PIP) 35 cm $H_2O$; positive end expiratory pressure (PEEP) 15 cm $H_2O$; fraction of inspired oxygen (FiO2) 1.0). The girl was comatosed, with a Glasgow Coma Scale (GCS) of 3, and had fixed, isocoric pupils. An aEEG revealed a bilateral flat trace (pattern recognition) without seizures. Over the subsequent 26 hours, global heart failure with

poor pumping function and arterial hypotension developed. The decision was therefore taken to administer levosimendan (0.2 µg/kg/min); dobutamine (15 µg/kg/min); milrinone (0.7 µg/kg/min); norepinephrine (max. 0.4 µg/kg/min); and vasopressin (max. 1.3 mIU/kg/min). The MAP continued to fluctuate between 35-40 mmHg, and showed no improvement despite volume replacement.

To counter the increasing bilateral pulmonary infiltrates and left apical hyperinflation and thus improve oxygen saturation, the girl was appropriately repositioned. During the first 12 hours of the University Hospital of Bonn PICU admission, a SARS-CoV-2 infection was confirmed. A repeat chest X-ray revealed extensive bilateral pulmonary infiltrates consistent with COVID-19. A non-contrast chest CT showed extensive bilateral airspace consolidations, ground glass opacities secondary to pneumonia, and small bilateral pleural effusions. A cranial CT scan revealed mild diffuse cerebral edema, hyperdense internal veins, and bilateral thalami hypodensities. These signs were considered compatible with dural sinus thrombosis and venous infarction. The CSF was tested, and found to be negative for SARS-CoV-2.

Over the subsequent 12 hours, the ventilation parameters were intensified (PIP 38 mmHg, PEEP 18 mmHg, and FiO2 1.0). However, oxygenation remained insufficient (transcutaneous saturation level of around 90%). Radiologically, a slight increase in right-sided infiltrates and persistent pulmonary congestion were evident. Due to signs of dorsal bilateral pulmonary infiltrates on the CT, the girl was placed in a prone position. This led to a short-term improvement in ventilation, with a saturation level of up to 100% being achieved. However, her pulmonary status subsequently deteriorated, with a maximum lactate of 17.6 mmol/l and an increasing negative base excess, despite continuous buffering with sodium bicarbonate and a stable pH of 7.3. Ventilation was further intensified, and ECMO was discussed. However, ECMO was considered to be contraindicated by the cranial CT findings. The girl's condition

continued to deteriorate, and death occurred 62 hours post initial hospital-admission (8 days after the first symptoms of a viral infection).

## Human leukocyte antigen (HLA) typing from exome data

HLA-I genotypes were reconstructed using the exome data of the index patient. This was achieved: 1) using OptiType, in order to obtain HLA-I genotypes in a four-digit-resolution; and 2) by comparing the binding prediction of SARS-CoV-2 peptides with a human leukocyte antigen in silico susceptibility map.[1] The generated HLA-I genotypes, and the corresponding number of putatively binding peptides of SARS-CoV-2, are presented in Supplementary Data 1. With the exception of B*51:01, all inferred HLA-I genotypes are predicted to have sufficient binding affinities to a medium number of peptides. Thus, these findings do not indicate any specific combination of MHC class I genes that might explain a susceptibility to a more severe COVID-19 disease course.

## Description of Supplementary Data 1

Supplementary Data 1 contains three tables: The frist table lists rare variants fulfilling the criteria of both prioritization categories 1) and 2) (see main manuscript). The second table contains the genes that were used for priorization of variants. The third table provides an overview of HLA-I genotypes and the putative number of SARS-CoV-2 peptides binding to the respective allele with sufficient affinity, as estimated via an in silico study.

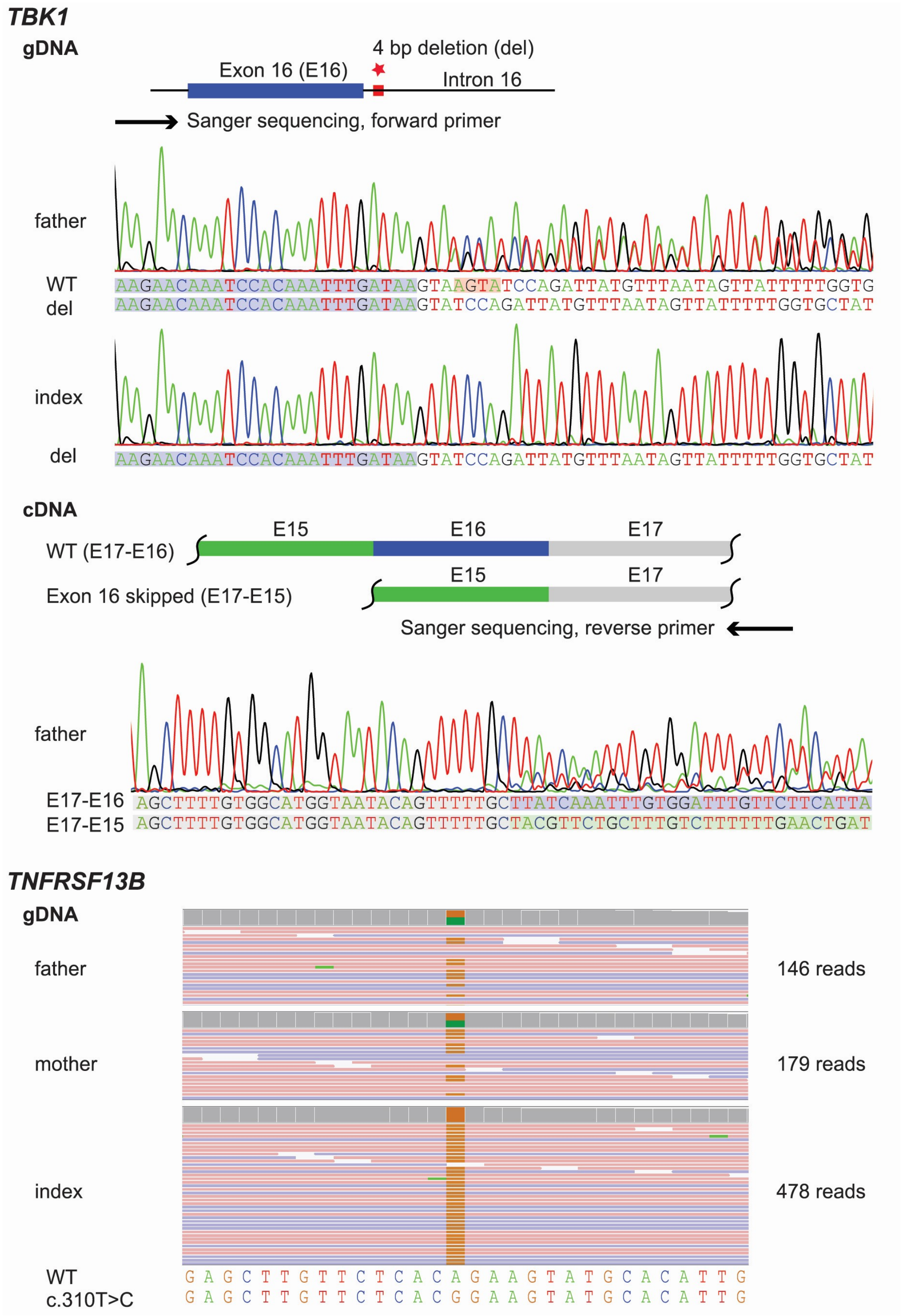


**Supplementary Figure 1: Raw sequencing results of the reported variants:** *TBK1*, upper panel: Sanger sequencing of genomic DNA (gDNA) from the father and the index patient. The 4 base pairs (NM_013254.4:c.1760+4_1760+7del) deleted in the variant allele are highlighted in red; exon 16 (E16) is highlighted by a blue bar. The sequence of the wild type

allele (WT) and the allele carrying the 4 bp deletion (del) are indicated below the respective chromatograms. *TBK1*, lower panel: Analysis of the complementary DNA (cDNA) of the father by Sanger sequencing. E15, E16, and E17 correspond to exon 15, exon 16, and exon 17, respectively. The WT sequence (E17-E16) and the sequence with a skipping of exon 16 (E17-E15) (r.1721_1760del) are indicated below the chromatogram. For *TBK1* gDNA and cDNA analysis, the results in the mother were virtually identical to those in the father, and are therefore not shown for the sake of brevity. *TNFRSF13B*: next-generation sequencing raw data for variant NM_012452.2:c.310T>C;p.(Cys104Arg), with sequencing reads colored according to the respective read strand.

**Supplementary References**

1. Nguyen, A. *et al*. Human Leukocyte Antigen Susceptibility Map for Severe Acute Respiratory Syndrome Coronavirus 2. *Journal of virology* **94** (2020).
