# Supplementary material for: TBK1 and TNFRSF13B mutations and an autoinflammatory disease in a child with lethal COVID-19
Source: NPJ Genom Med. 2021 Jul 1;6:55. doi: 10.1038/s41525-021-00220-w (PMC8249618; doi:10.1038/s41525-021-00220-w)
Supplement: Supplementary file 3 — Reporting Summary [file 41525_2021_220_MOESM3_ESM.pdf]

nature research

Corresponding author(s): Markus M. Nöthen

Last updated by author(s): Apr 6, 2021

Reporting Summary

Nature Research wishes to improve the reproducibility of the work that we publish. This form provides structure for consistency and transparency in reporting. For further information on Nature Research policies, see our Editorial Policies and the Editorial Policy Checklist.

Statistics

For all statistical analyses, confirm that the following items are present in the figure legend, table legend, main text, or Methods section.

n/a	Confirmed	
☒	☐	The exact sample size (*n*) for each experimental group/condition, given as a discrete number and unit of measurement
☒	☐	A statement on whether measurements were taken from distinct samples or whether the same sample was measured repeatedly
☒	☐	The statistical test(s) used AND whether they are one- or two-sided<br>*Only common tests should be described solely by name; describe more complex techniques in the Methods section.*
☒	☐	A description of all covariates tested
☒	☐	A description of any assumptions or corrections, such as tests of normality and adjustment for multiple comparisons
☒	☐	A full description of the statistical parameters including central tendency (e.g. means) or other basic estimates (e.g. regression coefficient) AND variation (e.g. standard deviation) or associated estimates of uncertainty (e.g. confidence intervals)
☒	☐	For null hypothesis testing, the test statistic (e.g. *F*, *t*, *r*) with confidence intervals, effect sizes, degrees of freedom and *P* value noted<br>*Give P values as exact values whenever suitable.*
☒	☐	For Bayesian analysis, information on the choice of priors and Markov chain Monte Carlo settings
☒	☐	For hierarchical and complex designs, identification of the appropriate level for tests and full reporting of outcomes
☒	☐	Estimates of effect sizes (e.g. Cohen's *d*, Pearson's *r*), indicating how they were calculated

Our web collection on statistics for biologists contains articles on many of the points above.

Software and code

Policy information about availability of computer code

Data collection	BWA version 0.7.17-r1188, GATK version 4.1.3.0.
Data analysis	Ensembl Variant Effect Predictor (VEP, version 97), VASE (version 0.24, https://github.com/david-a-parry/vase). No previously unreported computer code or algorithm was used. Data analysis was performed using standard tools, as described.

For manuscripts utilizing custom algorithms or software that are central to the research but not yet described in published literature, software must be made available to editors and reviewers. We strongly encourage code deposition in a community repository (e.g. GitHub). See the Nature Research guidelines for submitting code & software for further information.

Data

Policy information about availability of data

All manuscripts must include a data availability statement. This statement should provide the following information, where applicable:

- Accession codes, unique identifiers, or web links for publicly available datasets
- A list of figures that have associated raw data
- A description of any restrictions on data availability

A list of the genes that were prioritized for exome analysis is available in supplementary table 1, and the variants with the highest prioritization in our approach are provided in Supplementary Table 2. The chromatogram files underlying Supplementary Figure 1 are available upon request. The full exome data set cannot be shared due to a lack of parental consent. Variants were submitted to ClinVar, and are available under accession codes VCV000005302 and [submission code SUB9433729, will be replaced by accession code].

Field-specific reporting

Please select the one below that is the best fit for your research. If you are not sure, read the appropriate sections before making your selection.

☒ Life sciences ☐ Behavioural & social sciences ☐ Ecological, evolutionary & environmental sciences

For a reference copy of the document with all sections, see nature.com/documents/nr-reporting-summary-flat.pdf

Life sciences study design

All studies must disclose on these points even when the disclosure is negative.

Sample size	One single case with an extreme phenotype.
Data exclusions	The plausibility of calls of genetic variants was checked in the sequence alignments by persons experienced in exome evaluation. Variant calls that were regarded as being likely false positives were excluded from further analysis. The exome evaluators had gained their experience before conducting the current study in diagnostic exome sequencing.
Replication	Genetic variants that were not strongly supported by the next-generation sequencing data were confirmed by Sanger sequencing (here Sanger sequencing was performed for the variant in TBK1).
Randomization	No experimental groups were available, so randomization was not be performed.
Blinding	The investigators were not blinded.

Reporting for specific materials, systems and methods

We require information from authors about some types of materials, experimental systems and methods used in many studies. Here, indicate whether each material, system or method listed is relevant to your study. If you are not sure if a list item applies to your research, read the appropriate section before selecting a response.

Materials & experimental systems

n/a	Involved in the study	
☒	☐	Antibodies
☒	☐	Eukaryotic cell lines
☒	☐	Palaeontology and archaeology
☒	☐	Animals and other organisms
☐	☒	Human research participants
☒	☐	Clinical data
☒	☐	Dual use research of concern

Methods

n/a	Involved in the study	
☒	☐	ChIP-seq
☒	☐	Flow cytometry
☒	☐	MRI-based neuroimaging

Human research participants

Policy information about studies involving human research participants

Population characteristics	One female subject of Turkish ancestry.
Recruitment	This case report was initiated by the team of the Pediatric Intensive Care Unit of the University Hospital Bonn.
Ethics oversight	Our case report is a retrospective report of a clinical case and its diagnostic work-up. Thus, the report is not part of a planned scientific study. The relevant ethics committee of the University of Bonn was informed about the planned publication of the case report and has approved the procedure.

Note that full information on the approval of the study protocol must also be provided in the manuscript.
